# In-stent stenosis in the patient with internal carotid aneurysm after treated by the Willis covered stent: Two case reports and literature review

**DOI:** 10.1097/MD.0000000000006101

**Published:** 2017-02-17

**Authors:** Lun-Xin Liu, Meng-Yuan Song, Xiao-Dong Xie

**Affiliations:** a Department of Neurosurgery, West China Hospital; b School of Basic Science and Forensic Medicine, Sichuan University, Chengdu, Sichuan, People's Republic of China.

**Keywords:** covered stent, endovascular treatment, internal carotid artery aneurysm, stenosis

## Abstract

In-stent stenosis after treated by Willis covered stent—case reports.

**Background:**

Advancements in minimally invasive technology have allowed endovascular reconstruction of internal carotid aneurysm. However, in-stent stenosis is an important and well-characterized complication of stenting after the treatment of internal carotid aneurysm.

**Case description:**

We would present 2 patients who were diagnosed with in-stent stenosis after the treatment of Willis covered stent. Case 1: A 57-year-old female with 2-week history of headache and vomiting before admission, whose digital subtraction angiography (DSA) demonstrated left internal carotid C6 aneurysm and showed about 20% stenosis 3 months later since operation in the position where Willis covered stent was deployed. Case 2: A 23-year-old male with skull base fracture, subarachnoid hemorrhage, right femoral fracture for 14 days and epistaxis for 9 hours caused by a car accident, whose DSA demonstrated left internal carotid paracliniod pseudoaneurysm. One year later, the patient went to our center again because he had headache and dizziness for 6 months after the interventional operation. His DSA demonstrated about 80% stenosis in the position where Willis covered stent was deployed. The clinical and radiologic characteristics and the experience in dealing with the stenosis are presented.

**Conclusions:**

In-stent stenosis after treated with Willis covered is uncommon, but not rare. Operators should pay more attention to the in-stent stenosis during the period of follow-up observation and monitor P2Y12 Reaction Unit (PRU) in the antiplatelet period, especially for the Willis covered stent. What is more, the treatment for stenosis ought to be carefully considered.

## 1 Introduction

However, the International Subarachnoid Aneurysm Trial (ISAT) has proven that the endovascular treatment of cerebral aneurysms with detachable coils is a superior alternative to open microsurgery in terms of survival free of disability at 1 year, the recanalization rate of endovascular treatment is higher than the open microsurgery which is still a serious problem to be solved.^[1,2]^ What is more, aneurysm located in internal carotid artery (ICA) is difficult to deal with open microsurgery due to the bony obstacles and difficulty in proximal control.^[3–5]^ So that we should find a better endovascular technique to treat the aneurysm, especially the large or giant, complicated aneurysm or pseudoaneurysm, located in the ICA.

A novel stent was deployed in the parent artery to exclude the ICA aneurysm from circulation. Willis covered stent (MicroPort, Shanghai, China), a specifically designed balloon-expanded stent used in the intracranial vasculature, consists of 3 parts: a bare stent, an expandable polytetrafluoroethylene (ePTFE) membrane, and a balloon catheter.^[6–9]^ However, in-stent stenosis is not rare, as covered stents are more thrombogenic than others. In our center, 20 patients with ICA aneurysm received the treatment of Willis covered stent from August 6, 2014 to December 23, 2015 and only 2 were diagnosed with in-stent stenosis. One was asymptomatic with about 20% stenosis who received conservative treatment and the other was about 80% stenosis after digital subtraction angiography (DSA) diagnosis who used stent to resolve this problem. Written informed consent was obtained from both patients for the publication of their case reports and relevant images.

## 2 Case report

### 2.1 Case 1

**Figure 1:**
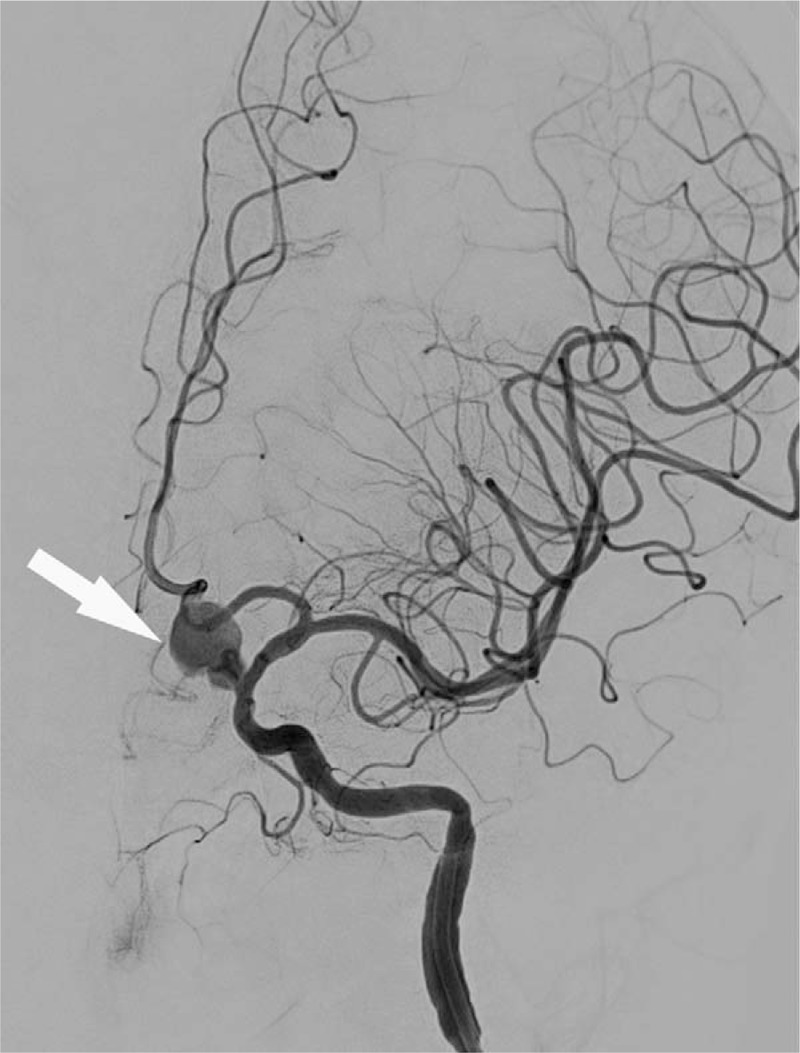
The white arrow demonstrated the aneurysm located at internal carotid artery C6.

A 57-year-old female with 2-week history of headache and vomiting before admission. Her physical examination showed neck stiffness, Glasgow Coma Scale (GCS) score was 15 points, head computed tomography (CT) revealed subarachnoid hemorrhage and DSA demonstrated left internal carotid C6 aneurysm (Fig. 1).

**Figure 2:**
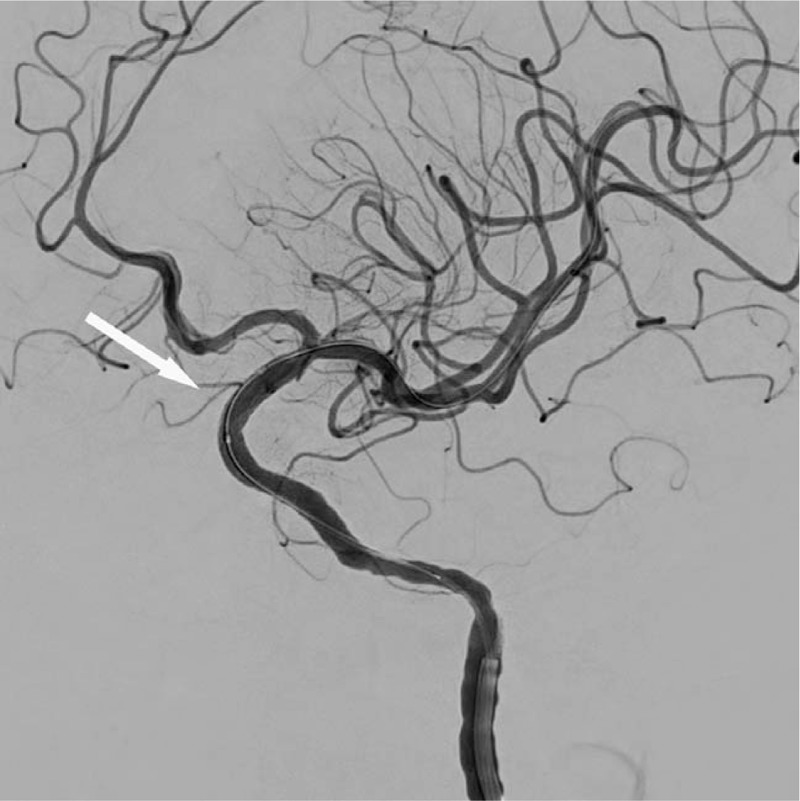
The white arrow showed the collapse of the aneurysm and satisfactory stent (a 3.5 mm × 10.0 mm Willis covered stent, MicroPort, Shanghai, China) positioning.

An endovascular reconstruction with Willis covered stent was scheduled. A Willis covered stent (3.5 mm × 10.0 mm, MicroPort) was deployed in the left internal carotid C6 segment. Intraoperative angiography demonstrated the collapse of the aneurysm and satisfactory stent positioning (Fig. 2).

**Figure 3:**
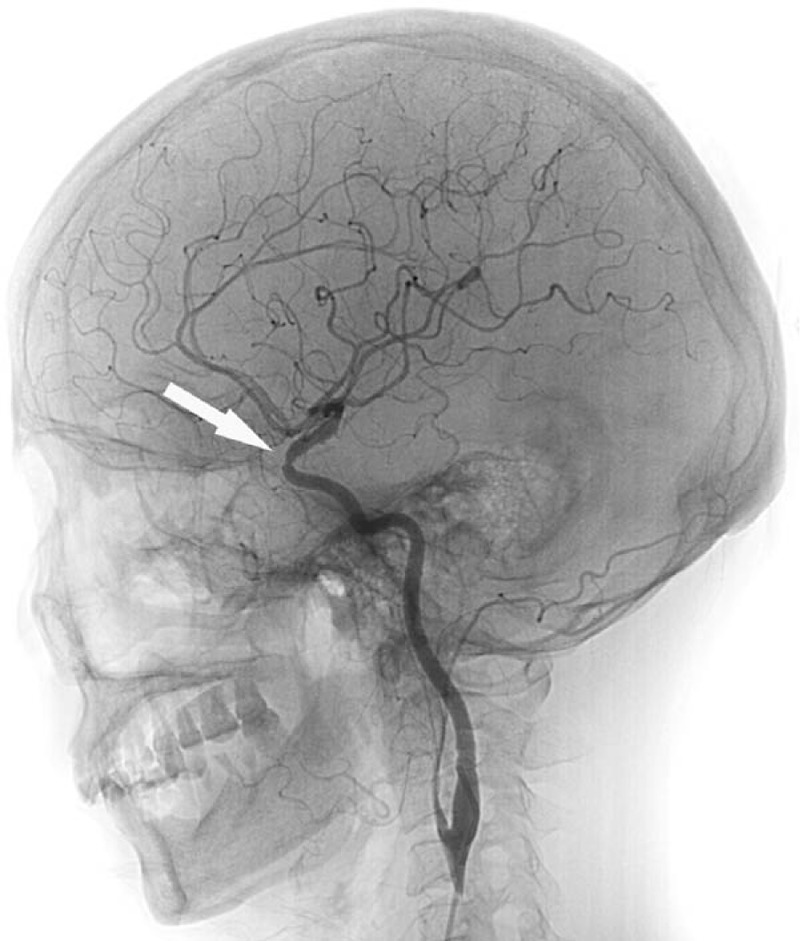
About 20% of stenosis in the site where deployed the Willis covered stent (white arrow) 3 months ago.

Three months later since operation, her DSA showed about 20% stenosis in the position where Willis stent was deployed (Fig. 3). As she was asymptomatic, we did not deal with it. Now she is still under our observation.

### 2.2 Case 2

**Figure 4:**
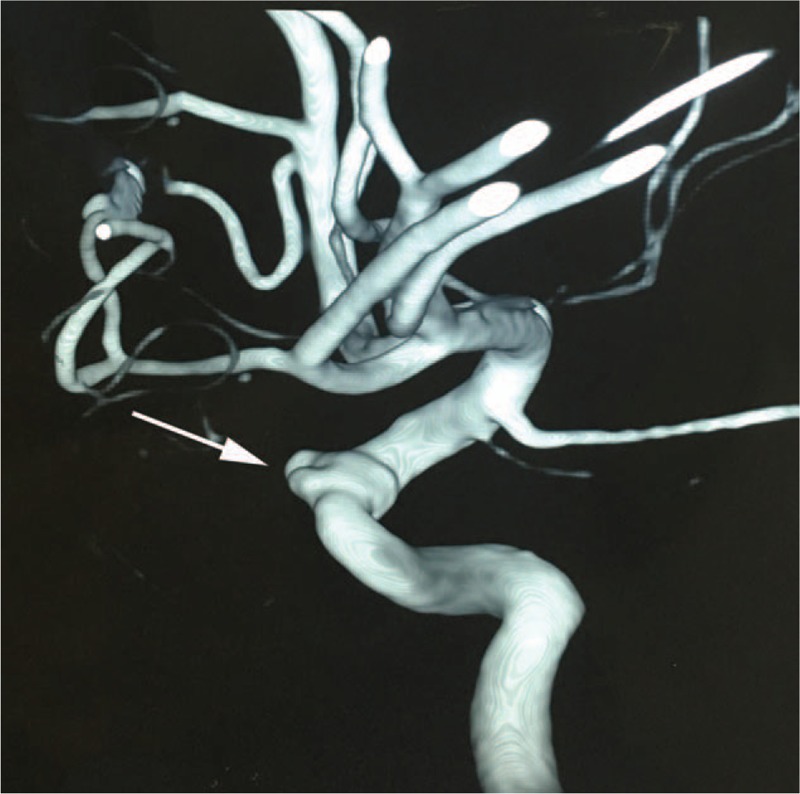
The white arrow showed the left internal carotid paracliniod pseudoaneurysm.

A 23-year-old male with skull base fracture, subarachnoid hemorrhage, right femoral fracture for 14 days and epistaxis for 9 hours caused by a car accident. Gauze packing and blood transfusion were used to prevent epistaxis. His physical examination revealed right leg movement restriction due to binding up his leg, his GCS score was 15 points, head CT demonstrated skull base fracture and subarachnoid hemorrhage, and X-ray showed right femoral fracture. After his admission to our center, his DSA demonstrated left internal carotid paracliniod pseudoaneurysm (Fig. 4).

**Figure 5:**
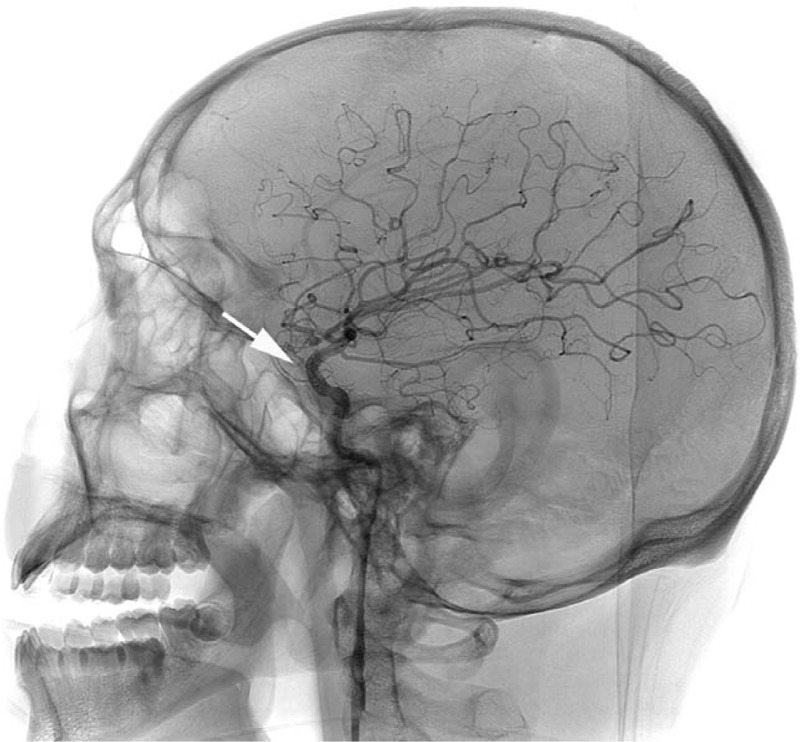
Intraoperative angiography showed the collapse of the pseudoaneurysm and a satisfactory stent (a 3.5 mm × 13 mm Willis covered stent, MicroPort, Shanghai, China) positioning (white arrow).

Before surgery, we gave him 225 mg clopidogrel and 300 mg aspirin for oral administration. Under the condition of general anesthesia and full heparinization, a Willis covered stent (3.5 mm × 13 mm) was deployed in the left internal carotid paracliniod segment. Intraoperative angiography showed the collapse of the pseudoaneurysm and a satisfactory stent positioning (Fig. 5). Three days later, this patient discharged.

**Figure 6:**
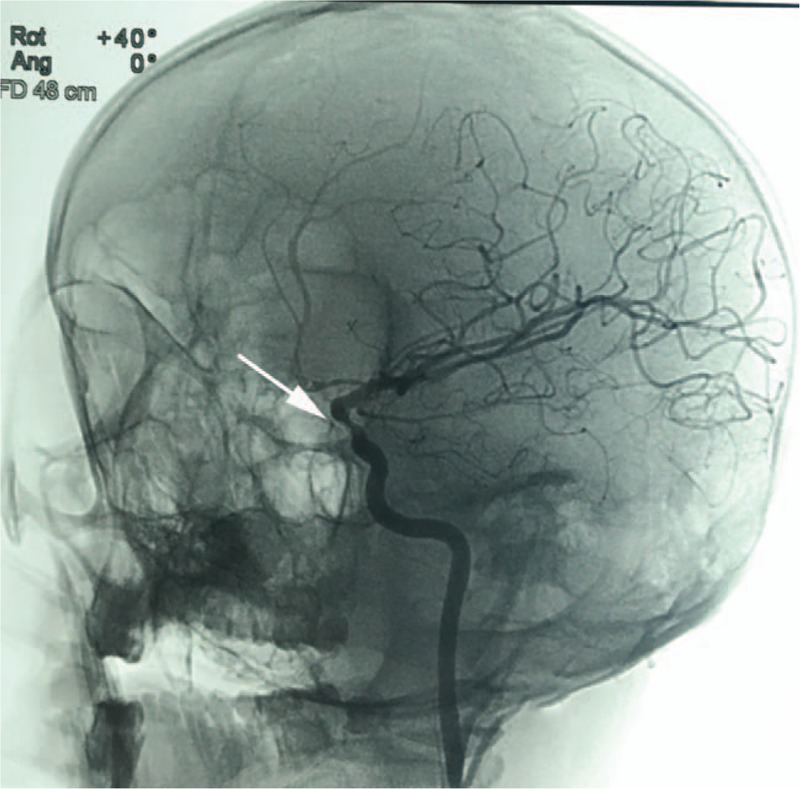
About 80% of stenosis in the position where deployed the Willis covered stent (white arrow).

**Figure 7:**
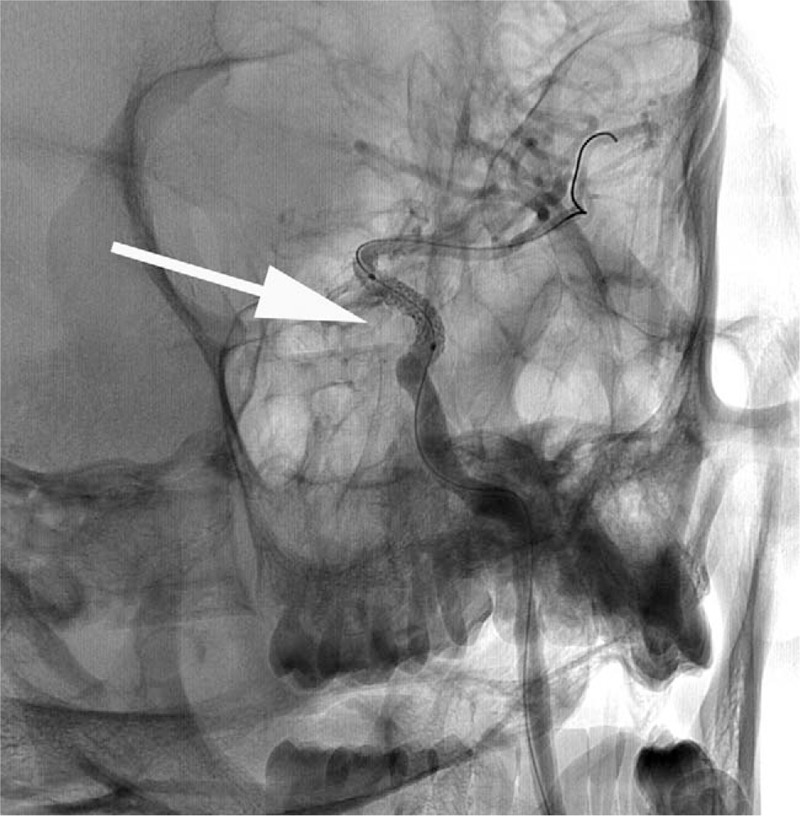
Intraoperative angiography demonstrated that the stenosis disappeared and that the left internal carotid artery kept patency after the stent deployed (Everolimus-Eluting Coronary stent 4.0 mm × 12 mm, Boston Scientific, Boston, USA).

One year later, he went to our center again as he had headache and dizziness for 6 months. His physical examination showed no abnormality and DSA diagnosis found about 80% stenosis in the position where Willis covered stent was deployed (Fig. 6). Everolimus-Eluting Coronary stent (4.0 mm × 12 mm, Boston Scientific, Marlborough, Massachusetts) was used to solve this problem. Intraoperative angiography demonstrated the stenosis disappeared and the left ICA kept patency (Fig. 7).

## 3 Discussion

Although the Wills novel covered stent has advantages in treating the complicated, wide necked, large, or giant aneurysms in ICA, its disadvantages should also be considered. Compared with other stents, in-stent stenosis is not rare, particularly in this reconstruction treatment technique (Table 1).^[2,10,11]^ The deployment of a balloon-expanded stent will inevitably result in endothelial disruption and denudation over the treated vascular segment. There will be a proliferation and activation of regional smooth muscle cells in the disappearance of functional endothelium, which will lead to neointimal tissue formation, finally resulting in in-stent stenosis.^[11,12]^ One important factor for the development of stenosis might be the reendothelialization of the stent area. Stent covering may lead to a prolonged process for reendothelialization as the middle of the stents had to be reached from stent edges. This longer time needed for reendothelialization could be a stimulus for more and higher proliferation of smooth muscle cell at stent edges because this process lasts as long as the endothelial layer is incomplete.^[13,14]^

**Table 1:**
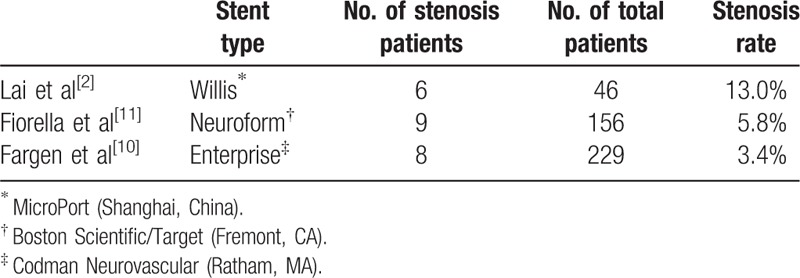
The stenosis rate of Willis covered stent compare with other kinds of stents.

As in-stent stenosis may spontaneously resolve along with the progress of natural history, the patient in the first case who had asymptomatic stenosis received conservative treatment.^[11]^ But the second case use an Everolimus-Eluting Coronary stent. In China, Everolimus-Eluting Coronary stent is much cheaper than Willis covered stent and pseudoaneurysm could be repaired by Willis covered stent, that is why we choose another kind of stent. In our center, the treatment is reserved for patients who develop ischemic symptoms during the follow-up observation, show new focus of asymptomatic ischemic injury on magnetic resonance imaging (MRI) within the ipsilateral vascular distribution, or appear a “steal phenomenon” on a cerebral blood flow study.^[11]^

According to the literature review, it was found that postprocedure irregular antiplatelet therapy and cerebrovascular arteriosclerosis, diabetes and lesion length, multiple stents, and smaller final minimal lumen diameter are associated with increased risk of in-stent stenosis.^[2,15,16]^ Both patients in the literature had no history of diabetes and cerebrovascular arteriosclerosis, and they were all deployed with only 1 Willis covered stent, aneurysm is in the trunk of the ICA. But the patient in Case 2 received irregular antiplatelet therapy, and that may be the reason why he developed in-stent stenosis.

The patient in Case 1 received regular antiplatelet therapy, but she still developed in-stent stenosis. Prabhakaran et al^[17]^ found that aspirin resistance was relatively uncommon, whereas clopidogrel resistance occurred in half of patients undergoing cerebrovascular stent placement. And P2Y12 Reaction Unit (PRU) is changeable in the same patient during the antiplatelet procedure.^[18]^ Maybe the patient in Case 1 is clopidogrel resistance, she ought to be monitored the PRU in the antiplatelet period. Furthermore, cigarette smoking and use of medications (proton pump inhibitors, antifungal or antihuman immunodeficiency virus medications, and antidepressants may influence the clopidogrel resistance).^[19]^

## 4 Conclusion

In-stent stenosis after treated with Willis covered is uncommon, but not rare. Operators should pay more attention to the in-stent stenosis in the follow-up observation period and monitor the PRU in the antiplatelet period, especially for the Willis covered stent. What is more, the treatment of stenosis ought to be carefully considered.
